# Cyclooxygenase-2-expressing macrophages in human pterygium co-express vascular endothelial growth factor

**Published:** 2011-12-29

**Authors:** Choul Yong Park, Jong Sun Choi, Sung Jun Lee, Sang Won Hwang, Eo-Jin Kim, Roy S. Chuck

**Affiliations:** 1 Department of Ophthalmology, Dongguk University Seoul, Graduate School of Medicine, Seoul, South Korea; 2 Department of Pathology, Dongguk University Seoul, Graduate School of Medicine, Seoul, South Korea; 3 Department of Ophthalmology and Visual Sciences, Montefiore Medical Center and Albert Einstein College of Medicine, Bronx, NY

## Abstract

**Purpose:**

To evaluate cyclooxygenase-2 (COX-2) expression and to characterize COX-2-expressing stromal cells in human pterygium.

**Methods:**

Primary pterygium tissue of Korean patients (eight males and nine females) was analyzed. The clinical characteristics were classified, and immunohistochemical staining using primary antibodies against cyclooxygenease-2, vascular endothelial growth factor-A, cluster of differentiation (CD)68, CD3, CD20, and leukocyte common antigen was performed.

**Results:**

COX-2 expression was detected in all pterygium tissues evaluated (17 primary pterygia). Diffuse expression of COX-2 in the epithelial layer was observed in nine samples. Infiltration of strongly positive COX-2 cells into the epithelial layer was a more common observation than diffuse epithelial COX-2 expression. Scattered COX-2-expressing cells in the stromal layer were found in all samples. Some COX-2-positive cells were found within microvessels. In addition to stromal COX-2-expressing cells, a few vascular endothelial cells strongly expressed COX-2; however most of the vessels were negative for COX-2 expression. Stromal COX-2-expressing cells were positive for the macrophage marker CD68 and co-expressed vascular endothelial growth factor. COX-2 expression in normal conjunctiva was not observed in seven control samples.

**Conclusions:**

These COX-2- and vascular endothelial growth factor-expressing macrophages may have relevance to the pathogenesis of pterygium.

## Introduction

Human pterygium is made up of chronic proliferative fibro-vascular tissue growing on the ocular surface. This disease exhibits both degenerative and hyperplastic properties [1,2]. Ultraviolet (UV)-light damage, dry and dusty environments, and repeated microtrauma can lead to development of pterygium in susceptible individuals [1,3,4]. Immunological mechanisms both humoral (Immunoglobulin [Ig] A, IgM, and IgG) and cellular (lymphocytes, plasma cells, and mast cells) are believed to play roles in pterygium development and recurrence [5-8]. Tumor-like characteristics of pterygium, such as virus infection by the likes of human papilloma virus, inactivation of tumor suppressor gene *p53*, and co-existence with ocular surface neoplasm, have been reported [9-11]. Possible roles of bone marrow progenitor cells and neuronal signals in pterygium have recently been suggested [12-14]. Hyper-vascularity is one of the characteristic cosmetic problems of pterygium and leads young patients to surgical removal of the lesion. Although the exact pathogenesis is still unclear, chronic inflammation, angiogenesis, and uncontrolled proliferation are the key features of pterygium [1,2,4,15]. Therefore, it is highly suspected that several inflammatory and angiogenic factors are closely related to its pathogenesis.

Cyclooxygenase-2 (COX-2) is an inducible isoform of cyclooxygenases and is the key enzyme for inflammatory cytokine-induced angiogenesis. Recently, COX-2 was reported to increase vascular endothelial growth factor (VEGF) expression in chronic inflammation and various tumors [16-20]. While cyclooxygenase-1 is constitutively expressed in most types of cells and tissues, COX-2 is rapidly induced by growth factors, cytokines, bacterial endotoxins, and tissue damage. COX-2 is also involved in the pathogenesis of skin tumors in conjunction with reactive oxygen species generated by UV damage. Recent studies indicate COX-2 expression in human pterygium and suggest its role in disease pathogenesis and prognosis after surgical excision [21-24].

Although several previous studies verified the existence of COX-2 expression in human pterygium, there has been no study to characterize these COX-2-expressing cells and investigate the correlation with VEGF. Therefore, the aim of this study is to investigate the characteristics of COX-2-expressing cells in pterygium. In addition to analyzing the spatial distribution of COX-2-expressing cells, various inflammatory cell markers were used to characterize them. Finally, the co-expression of COX-2 and VEGF was evaluated.

## Methods

Primary pterygium tissue was harvested after obtaining informed consent from Korean patients (eight males and nine females). All patients were diagnosed with primary pterygium in the nasal conjunctiva. None had been under topical medication treatment except for artificial tear drops. No sign of severe inflammation of pterygium was observed in any of the patients. Normal conjunctiva was harvested from the superior conjunctiva of seven patients (four males and three females, ages from 59 to 78 years) after obtaining informed consent when they underwent cataract surgery.

### Clinical classification of pterygium

The clinical characteristics of pterygium were classified using a modified classification system [25]. The stage of pterygium was rated as stage I, tissue involvement of limbus; stage II, tissue just on the limbus; stage III, tissue between the limbus and pupillary margin; and stage IV, tissue central to the pupillary margin. The surface vascularity (V) of pterygium was scored as score +, minimal visible vessel (equal to conjunctiva); score ++, moderate vascularity (more dense than conjunctiva); and score +++, severe vascularity with vessel congestion. Conjunctival tissue thickenss (C) was classified as C1, flat tissue; C2, minimally elevated tissue; C3, tissue elevation up to 1 mm; and C4, tissue elevation over 1 mm. Corneal tissue thickness (K) was classified as K1, flat tissue; K2, minimally elevated tissue; K3, tissue elevation up to 1 mm; and K4, tissue elevation over 1 mm. This study was performed with approval from the Institutional Review Board of Dongguk University Hospital, Koyang, South Korea.

### Immunohistochemical study

For immunohistochemical studies, 4-µ-thick sections were obtained from formalin-fixed, paraffin-embedded tissues and were transferred onto adhesive slides and dried at 60 °C for 40 min. Immunohistochemical procedures were performed using a BenchMark XT automatic immunohistochemical staining device (Ventana Medical System, Tucson, AZ). In brief, after dewaxing and rehydrating, antigen retrieval was performed using microwave technique (citrate buffer, pH 6.0, twenty-five min in a microwave oven). The slides were then incubated for 30 min at 42 °C with primary antibodies. The primary antibody was detected using iVIEW DAB detection kit (Ventana Medical System), which is highly specific and sensitive to rabbit and mouse immunoglobulins. The detailed information of the primary antibodies used in this study is provided in Table 1.

**Table 1:** Description of primary antibodies used in the study.

**Target**	**Description**	**Manufacturer**	**Catalog no.**
Cyclooxygenease-2	Rabbit polyclonal	Abcam	ab15191
Vascular endothelial growth factor-A	Mouse monoclonal	Abcam	ab1316
CD68	Mouse monoclonal	Dako	M0876
CD38	Mouse monoclonal	Dako	F7101
CD3	Rabbit polyclonal	Dako	A 0452
CD20	Mouse monoclonal	Dako	IR604
Leukocyte common antigen	Mouse monoclonal	Dako	M 0701
Alexa Fluor 594		invitrogen	A11012
Alexa Fluor 488		invitrogen	A11001

COX-2 expression in the epithelial layer was scored for the percentage of positive-staining cells: 0, negative staining; +, from 1 to 10%; ++, from 11 to 50%; and +++, more than 50% positive cells. COX-2 expression in the stromal layer was scored for the average number of COX-2-expressing cells calculated over ten random high-power fields (400×): 0, no positive cells; +, from one to five positive cells; ++, from six to ten positive cells; and +++, more than ten positive cells.

### Western blot analysis

For western blot analysis, the parts of pterygium tissues (case 2, 3, 4, 6, 10, and 16) stored at –80 °C were quickly homogenized with a tissue crusher while frozen, and the tissue powder was placed into boiling lysis buffer (1% SDS, 1.0 mM sodium ortho-vanadate, 10 mM Tris [pH 7.4]), placed into a microwave oven for 15 s, and centrifuged for 5 min at 11,400× g at 15 °C. The samples were then separated via SDS–PAGE under denaturing conditions and electroblotted onto a polyvinylidine difluoride membrane (BioRad, Hercules, CA). After being blocked using 5% nonfat dry milk in Tris-buffered saline containing 10 mM Tris (pH 7.6), 150 mM NaCl, and 0.1% Tween-20, the membranes were incubated with anti-COX-2 antibodies (catalog no: ab15191; Abcam, Cambridge, MA), and diluted to 1:200 in blocking solution overnight at 4 °C. The membranes were further incubated with an antirabbit horseradish peroxidase-conjugated antibody (Santa Cruz Biotechnology, Santa Cruz, CA). They were then treated with an enhanced chemiluminescence solution (Pierce Fast Western Blot kit; Thermo fisher Scientific, Rockford, IL), and the signals were captured on an image reader (Las-3000; Fuji Photo Film, Tokyo, Japan). To monitor the amount of protein loaded into each lane, the membranes were treated with a stripping buffer and re-probed with a monoclonal antibody against β-actin (Sigma-Aldrich, St Louis, MO). The protein bands were analyzed via densitometry. The ratio between COX-2 and β-actin was calculated.

### Statistical analysis

The χ^2^ test was used for statistical analysis. P values less than 0.05 were considered to be significant.

## Results

**Figure 1:**
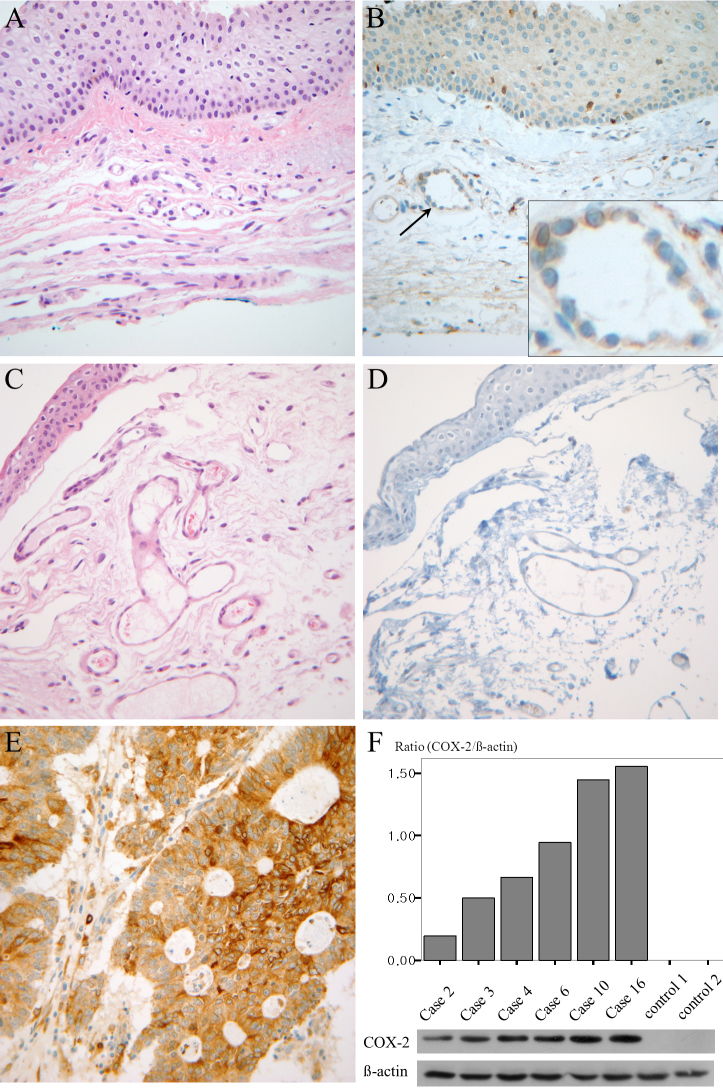
Cyclooxygenase-2 expression in pterygium tissue. **A**: Hematoxylin and eosin staining of human pterygium (case 10) is demonstrated. **B**: Immunohistochemistry of human pterygium using anti-cyclooxygenase-2(COX-2) antibody was performed. Diffuse expression (grade 2) of COX-2 is seen in the epithelial layer with some strong COX-2-expressing cells in both the epithelium and stroma. Endothelial cells lining vessels were also strongly positive for COX-2 expression in this sample. The arrow indicates the magnified area. **C**, **D**: COX-2 expression was not found in the epithelial or stromal layer in the normal control. **E**: Strong COX-2 expression in colon cancer was demonstrated as the positive control. **F**: western blot analysis revealed various degrees of COX-2 expression in pterygium tissue (cases 2, 3, 4, 6, and 10); however, no COX-2 band was observed in the control tissue.

**Figure 2:**
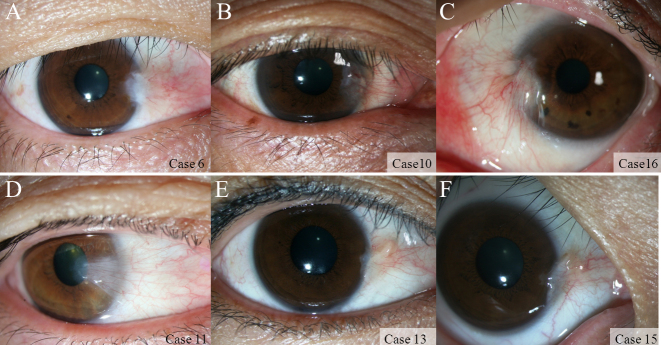
Anterior segment photographs of representative pterygium cases. Upper panels (**A**-**C**) demonstrate cases 6, 10, and 16. The surface vascularity of these pterygia was scored as +++. These cases revealed strong cyclooxygenase (COX-2) expression both in the epithelial and stromal layers. Lower panels (**D**-**F**) demonstrate cases 11, 13, and 15. These pterygia look slightly atrophic, and the vascularity was scored as ++. Relatively fewer COX-2-expressing stromal cells were found in these cases compared to cases of the upper panel. The surface vascularity of pterygium was scored as score +, minimal visible vessel (equal to conjunctiva); score ++, moderate vascularity (more dense than conjunctiva); and score +++, severe vascularity with vessel congestion.

**Figure 3:**
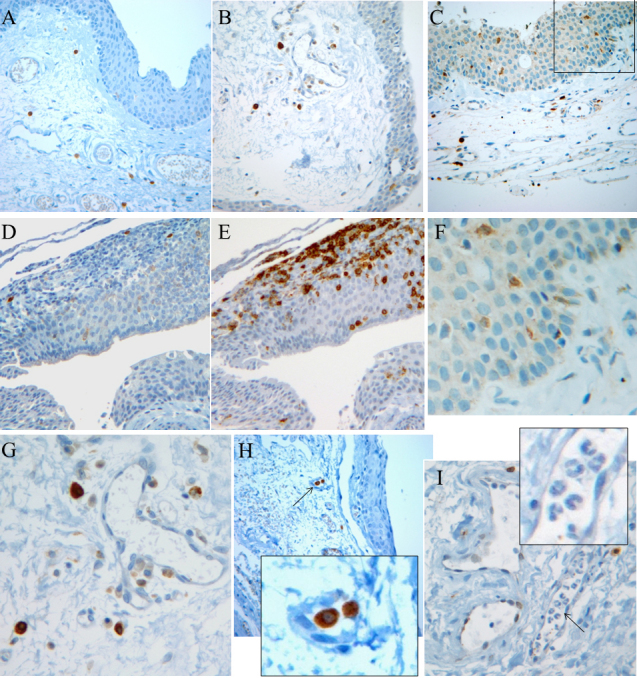
The various patterns of cyclooxygenase-2 expression in human pterygium. Panels **A** to **C** demonstrate score 0 (**A**, case 13), score ++ (**B**, case 1) and score +++ (**C**, case 16) expression of cyclooxygenase-2 (COX-2) in the epithelial layer of pterygium. Strong COX-2-expressing stromal cells were always observed. **D**: A few COX-2-expressing cells were found within a cluster of inflammatory cells in case 4. **E**: These inflammatory cells were mostly CD3-expressing T cells. **F**: The epithelial layer of panel **C** shows a few, scattered, strong COX-2-expressing cells. **G**: COX-2-expressing cells in panel **B** were magnified. These cells were found near blood vessels; however, endothelial cells were negative for COX-2 expression. Three cells show very strong positive COX-2 expression both in the cytoplasm and in the nucleus. **H**: Strong COX-2-expressing cells were localized within microvessels (case 15). The arrow indicates the magnified area. **I**: Inflammatory cells within vessels show segmented nuclei; however, these cells were negative for COX-2 expression (inserted image). A few endothelial cells express COX-2 (case 6). COX-2 expression in the epithelial layer was scored for the percentage of positive-staining cells: 0, negative staining; +, from 1 to 10%; ++, from 11 to 50%; and +++, more than 50% positive cells.

**Figure 4:**
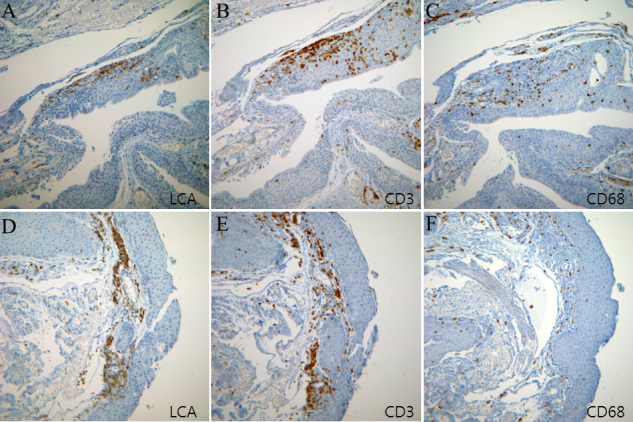
Lymphocytes and macrophages scattered throughout the pterygium. Immunohistochemistry using anti-leukocyte common antigen (LCA), anti-CD3, and anti-CD68 antibodies showed the characteristic distribution of inflammatory cells. **A**-**C**: Tissue image was obtained from case 4. **D**-**F**: Tissue image was obtained from case 8. LCA and CD3 staining showed a similar clustered distribution, suggesting most LCA-expressing cells are T cells. CD68-expressing cells are smaller in population and have an evenly scattered distribution.

**Figure 5:**
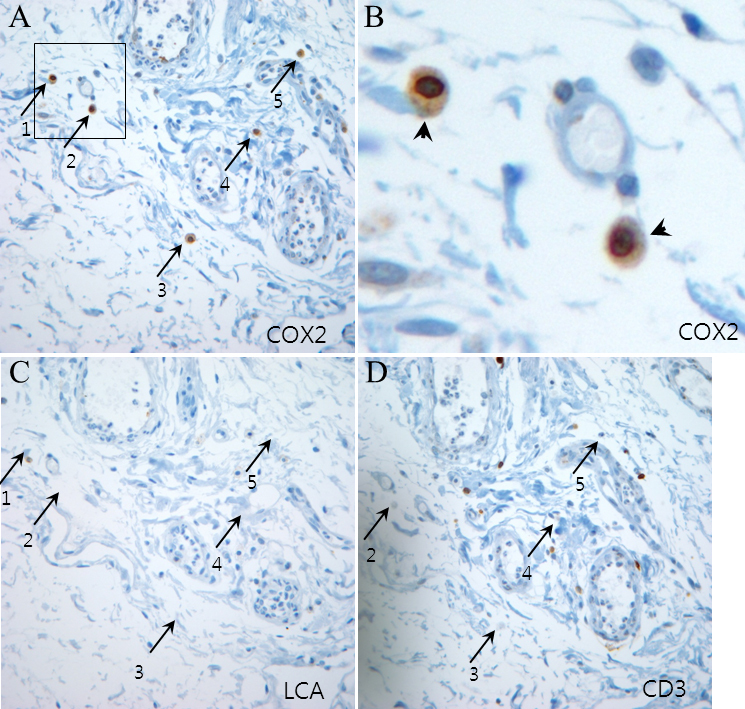
Expression of cyclooxygenase-2 (COX-2), leukocyte common antigen (LCA), and CD3. **A**: COX-2-expressing cells have round and oval shapes. **B**: A magnified view of the outlined area of panel **A**. COX-2-expressing cells have oval shapes and abundant cytoplasm. Strong COX-2 staining was observed both in the cytoplasm and in the nucleus. (arrowheads) **C**-**D**: Immunohistochemistry of adjacent sections of tissue with anti-LCA and anti-CD3 antibodies (T-cell marker) was performed. COX-2-expressing cells (arrows 1 to 5) showed different distributions compared to inflammatory cells expressing LCA or CD3 (case 7).

**Figure 6:**
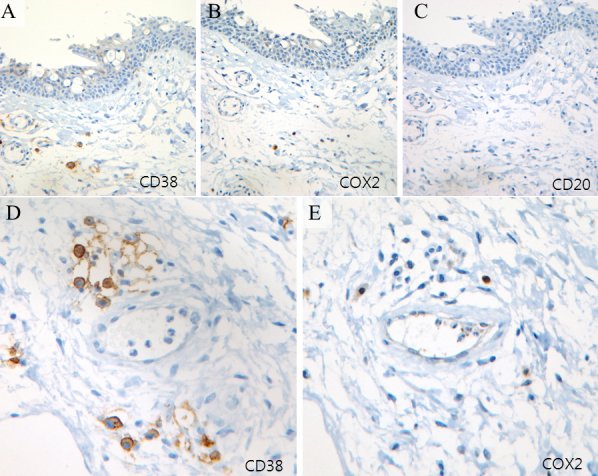
Expression of cyclooxygenase-2 (COX-2), CD38, and CD20. **A**-**C**: Immunohistochemistry of adjacent sections of tissue with anti-CD38, anti-COX-2, and anti-CD20 antibodies (B cell marker) was performed in case 11. CD38-expressing plasma cells were scattered through the stromal area; however, CD20-expressing cells were rare. **D**, **E**: CD38-expressing plasma cells were clustered around the blood vessel; however, COX-2-expressing cells showed different distributions from inflammatory cells expressing CD38.

**Figure 7:**
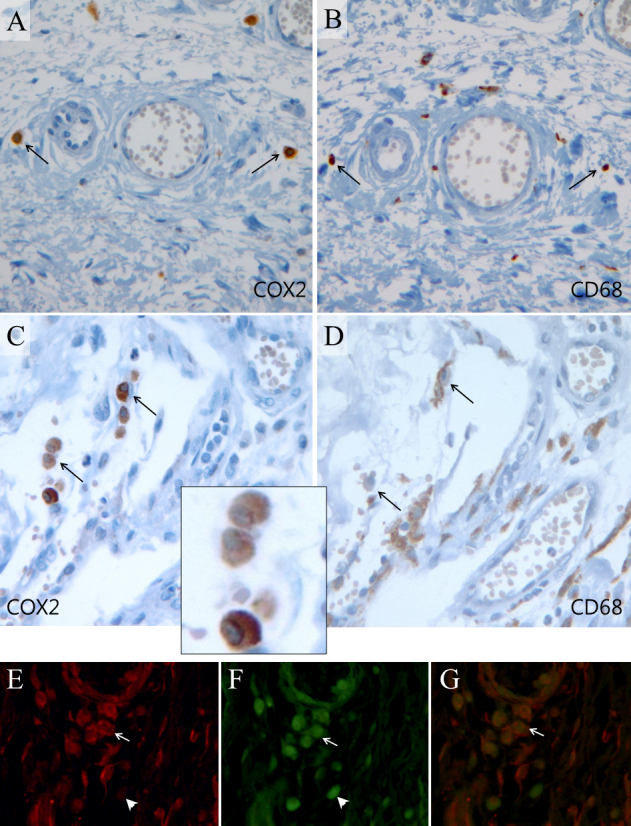
Expression of cyclooxygenase-2 (COX-2) and CD68. Immunohistochemistry of adjacent sections of tissue using anti-COX-2 antibody and anti-CD68 antibody was performed in case 9 (**A**, **B**), case 2 (**C**, **D**), and case 10 (**E**-**G**). Arrows indicate the cells co-expressing COX-2 and CD68. Not all CD68-expressing cells co-expressed COX-2; however, most COX-2-expressing cells co-expressed CD68. Panel **C** includes the magnified image of COX 2-expressing cells that have a morphology suggesting macrophages. Panels **E** and **F** are stained with fluorescent antibody targeting COX-2 (red) and CD68 (green). The merged image (panel **G**) shows that COX-2 and CD68 are expressed in the same cells (arrow). The arrowhead indicates one macrophage that expressed CD68 but not COX-2.

**Figure 8:**
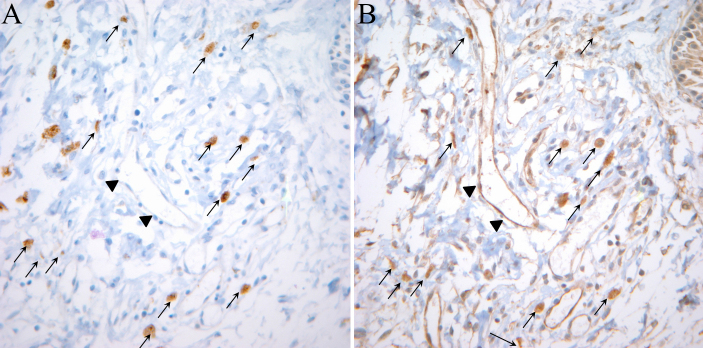
Co-expression of cyclooxygenase-2 (COX-2) and vascular endothelial growth factor (VEGF). Immunohistochemistry of adjacent sections of tissue using anti-COX-2 antibody (**A**) and anti-VEGF-A antibody (**B**) was performed in case 2. The arrows indicate cells co-expressing COX-2 and VEGF-A. VEGF-A-expressing vascular endothelial cells in this slide were negative for COX-2 expression. (arrowheads).

**Figure 9:**
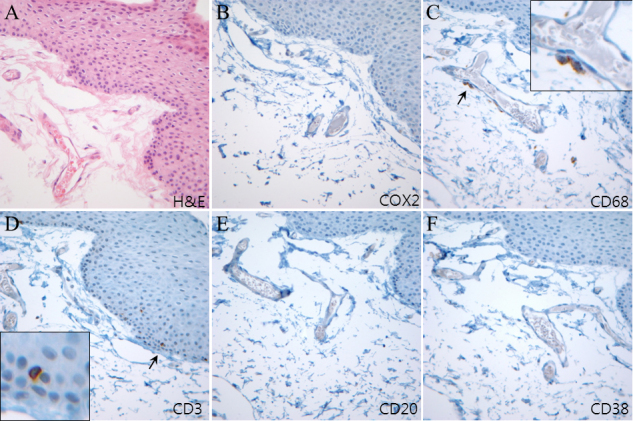
Immunohistochemistry of adjacent sections of normal conjunctiva (control). **A**: Hematoxylin and eosin staining is demonstrated. **B**: Cyclooxygenase-2 expression was not observed in either the epithelial or stromal layer. **C**: A few CD68-expressing macrophages were observed near blood vessels. The inserted image is the magnification of the arrow-indicated area. **D**: Intra-epithelial CD3-expressing T cells were observed. The inserted image is the magnification of the arrow-indicated area. **E**, **F**: Neither CD20-expressing cells nor CD38-expressing cells were found.

The clinical characteristics of the pterygium studied are listed in Table 2. COX-2 expression in the primary pterygium was verified by immunohistochemistry and western blot analysis. Diffuse expression of COX-2 in the epithelial layer was observed in nine samples (Table 2; Figure 1). Infiltration of strong COX-2-expressing cells into the epithelial layer was observed in samples showing diffuse epithelial COX-2 expression. Stromal COX-2 expression was more prominent in pterygia having prominent vascularity compared to pteygia having atrophic appearance (Figure 2). Scattered COX-2-expressing cells in the stromal layer were more common and were found in all samples (Figure 3). These COX-2-expressing stromal cells were readily detectable around blood vessels and were scattered rather than clustered. Some strongly expressing COX-2 cells were located within microvessels, suggesting that these cells were migrating from blood vessels (Figure 3H). Some vascular endothelial cells strongly expressed COX-2, as found in four samples (Figure 1B). However, most of the stromal vessel endothelial cells showed negative expression of COX-2 (13 samples; Figure 3G). Interestingly, four samples expressing endothelial COX-2 expressed higher grade (++ or +++) of epithelial COX-2 compared to other samples (p=0.002, χ^2^ test). Higher grade (+++) of stromal COX-2 expression was also more frequently found in these four samples (p=0.010, χ^2^ test). In addition, three samples of these (75%) were classified as stage III and V +++. Considering only 35% of studied samples showed both stage III and V +++, it is possible that vascular COX-2 expression is related to clinical severity, although statistical significance was not reached due to the small sample size (p=0.099, χ^2^ test). White blood cells with segmented nuclei were occasionally found in the stromal area but were negative for COX-2 expression (Figure 3I). T cells and macrophages were scattered throughout the pterygium. T cells were the largest population of inflammatory cells and showed a clustered pattern in distribution, whereas macrophages were small in population but showed evenly scattered distribution (Figure 4). Most of the COX-2-expressing stromal cells had round- to oval-shaped cytoplasm, and both nucleus and cytoplasm were strongly stained (Figure 5). The distribution pattern and the shapes of COX-2-expressing stromal cells raised the suspicion that these cells were infiltrating inflammatory cells. Subsequent staining with inflammatory cell markers (CD3, CD20, CD68, CD38, and leukocyte common antigen) revealed that the cells were CD68-expressing macrophages (Figure 5, Figure 6, Figure 7). Stromal COX-2-expressing cells co-expressed VEGF; however, VEGF-expressing vascular endothelial cells were mostly negative for COX-2 expression (Figure 8). COX-2 expression in normal conjunctiva was not observed in seven control samples (Figure 1, Figure 9).

**Table 2:** Clinical characteristics and cyclooxygenase-2 expression pattern of pterygia studied were demonstrated.

**Case No.**	**Sex/Age**	**Stage**	**V**	**C**	**K**	**COX-2 (epithelial layer)**	**COX-2 (stromal vessels)**	**COX-2 (stromal layer)**
1	M/52	III	+++	C3	K2	++	-	+++
2	F/53	III	++	C2	K2	0	-	+++
3	F/50	II	++	C3	K1	++	+	+
4	M/43	III	+++	C2	K2	+	-	++
5	M/57	II	++	C3	K2	0	-	++
6	M/39	III	+++	C3	K1	++	+	+++
7	F/53	II	++	C3	K2	0	-	++
8	F/33	III	+++	C2	K2	0	-	++
9	M/74	III	++	C2	K2	0	-	++
10	M/66	III	+++	C2	K2	+++	+	+++
11	F/47	III	++	C3	K2	+	-	+
12	M/59	III	++	C2	K2	0	-	++
13	F/52	II	++	C2	K1	0	-	+
14	M/60	III	++	C2	K2	+	-	++
15	F/47	II	++	C2	K1	0	-	+
16	F/53	III	+++	C3	K2	+++	+	+++
17	F/55	II	++	C2	K2	+	-	++

Stage: I, tissue involvement of limbus; II, tissue just on the limbus; III, tissue between the limbus and pupillary margin; and IV, tissue central to the pupillary margin. Surface vascularity (V): +, minimal visible vessel (equal to conjunctiva); ++, moderate vascularity (more dense than conjunctiva); and +++, severe vascularity with vessel congestion. Conjunctival tissue thickenss (C): C1, flat tissue; C2, minimally elevated tissue; C3, tissue elevation up to 1mm; and C4, tissue elevation over 1mm. Corneal tissue thickness (K): K1, flat tissue; K2, minimally elevated tissue; K3, tissue elevation up to 1mm; and K4, tissue elevation over 1mm. COX-2 expression in the epithelial layer: score 0, negative staining; score +, from 1 to 10%; score ++, from 11 to 50%; and score +++, more than 50% positive cells. COX-2 expression in the stromal layer: score 0, no positive cells; score +, from 1 to 5 positive cells; score ++, from 6 to 10 positive cells; and score +++, more than 10 positive cells.

## Discussion

Several theories have been suggested concerning the pathogenesis of human pterygium. The observed heavy infiltration of inflammatory cells and immunoglobulins has been suggested as evidence for an immune process in pterygium pathogenesis [1,2,4]. On the other hand, COX-2 expression and attenuation of p53 along with UV-light damage has suggested tumor-like characteristics of pterygium [1,2,4,26]. Degenerative change and stem cell involvement have also been verified in previous studies [1,4,12].

COX-2 expression in human pterygium has been previously studied. Chiang et al. found that more than 80% of pterygium tissue studied showed positive expression of COX-2 in the epithelial layer; however, they found no COX-2 expression in the stromal layer [22]. Subsequent studies by Karahan et al. [23] and Maxia et al. [24] reported COX-2 expression in the epithelial layer of primary pterygia, at 84.2% and 67.7%, respectively. They also reported COX-2 expression in the stromal layers. Capillaries, inflammatory cells, and stromal cells were positive for COX-2 expression in these studies. Strong COX-2 expression was suggested as a risk factor for recurrence of pterygium [21,23]. In our study, nine out of 17 pterygia (52.9%) expressed COX-2 in the epithelial layer and 100% expressed COX-2 in the stromal layer, especially in macrophages infiltrating the lesion. The heterogeneity of epithelial COX-2 expression may be related to the inflammatory state of the ocular surface; however, the uniform finding of stromal COX-2-expressing cells and increased COX-2 expression in pterygia having more prominent clinical appearance suggests that these cells are closely related to the pathogenesis or maintenance of pterygium. These COX-2-expressing macrophages were also found to infiltrate the epithelial layers.

Normal conjunctiva contains a local surface immune system called conjunctiva-associated lymphoid tissue (CALT) that contains T cells, B cells, and plasma cells as well as local secretions of immunoglobulins (IgA, IgM, IgG, and IgE) and complement [6,8,27,28]. This local immune system is not homogeneously distributed in the whole conjunctiva. Knop et al. [27] reported that CALT is more developed and populated in tarsal, orbital, and fornix conjunctiva. Bulbar conjunctiva was found to harbor relatively few CALTs compared to other parts of the conjunctiva. However, previous studies indicated that both immune cells and immunoglobulins are pathologically increased in pterygium tissue, which indicates that the immunologic process might be involved in its pathogenesis [6-8,29]. Although T cells are the most frequently encountered type of inflammatory cell [6,7], CD68-positive macrophages are the next most commonly encountered in pterygium and they are distributed in both the epithelial and stromal layers, suggesting that they are related to disease pathogenesis. Additionally in the current study, we found that some of these macrophages strongly expressed COX-2 and VEGF in pterygium.

COX-2 is one of the key molecules in the induction of angiogenesis in tumors [30]. Co-expression of COX-2 and VEGF in several tumors has been previously reported [17,31-33]. Exogenous addition of COX-2 protein can upregulate VEGF production via the protein kinase C pathway in lung cancer cells [20]. Prostaglandin E2, the product of COX-2 activity, is also angiogenic by direct influence on endothelial cells or by inducing the release of angiogenic growth factors, such as urokinase plasminogen activator receptor, fibroblast growth factor receptor-1, and VEGF [34]. VEGF is a potent stimulator of new vessels on the ocular surface. In the presence of VEGF, endothelial cells proliferate, migrate, and eventually form microvessels. Many studies have revealed the importance of VEGF in the pathogenesis of pterygium [1,4,26,35]. In our study, most VEGF expressing micro-vessels in stromal layer was negative for COX-2 expression. This result suggests that co-expression of COX-2 and VEGF in the same cell is not prerequisite for VEGF expression. Both COX-2 and VEGF can be therapeutic target in the treatment of pterygium. Anti-angiogenesis therapy using anti-VEGF antibodies has recently been reported to be effective in both controlling primary pterygium and preventing recurrence after pterygium excision [26,36-39]. And there was a report that selective COX-2 inhibitor (Nimesulide) inhibited the proliferation of pterygium fibroblast in dose dependent manner [40].

The finding that two important inflammatory molecules, COX-2 and VEGF, are highly expressed in macrophages infiltrating human pterygium suggests the critical role of these macrophages in pathogenesis. Macrophage expression of COX-2 was previously reported in several inflammatory disease and tumors [41-45]. In addition, recent studies have demonstrated that VEGF expression in macrophages is important in fibrosis and angiogenesis on the ocular surface [46,47]. Therefore, macrophages expressing both COX-2 and VEGF may have the potential to promote angiogenesis, collagen deposition, and possibly pterygium growth. Additionally, COX-2 and VEGF expression in macrophages suggest a role for these cells as a bridge between the immune process and the tumor-like characteristics of pterygium because both COX-2 and VEGF are important in skin carcinogenesis induced by UV-light damage [48,49].

There is a limitation of this study. A small sample size (n=17) of this study failed to provide significant evidence of correlation between vascular endothelial COX-2 expression and clinical severity. Future investigations using other modalities such as cell models may provide stronger evidence.

In summary, the present study reveals a population of macrophages expressing both COX-2 and VEGF in human pterygium. Despite advances in the treatment and management, the exact pathogenesis of pterygium is still unknown. Considering the immunologic and tumor-like characteristics of the disease, further studies about macrophages expressing both COX-2 and VEGF may provide a more comprehensive understanding about human pterygium.
